# Temperature Dependence of the Dynamic Parameters of Contact Thermometers

**DOI:** 10.3390/s19102299

**Published:** 2019-05-18

**Authors:** Silke Augustin, Thomas Fröhlich

**Affiliations:** Institute for Process Measurement and Sensor Technology, Technische Universität Ilmenau, G.-Kirchhoff-Str. 1, 98693 Ilmenau, Germany; thomas.froehlich@tu-ilmenau.de

**Keywords:** thermometer, dynamic, material properties, temperature dependence

## Abstract

Contact thermometers are used in a wide temperature range as well as under various media and environmental conditions. The temperature can range from −200 °C to about 1500 °C. In this case, the dynamic parameters (time percentage values *t_x_* and time constants *τ*) depend on temperature. Several effects are superimposed. Constructional and material properties of the thermometer and the installation location affect the dynamic behavior as well as the type and material properties of the object to be measured. Thermal conductivity *λ*, specific heat capacity *c*, and density *ρ* depend on temperature. This temperature dependence can be mutually compensated for (see Section 3). At the same time, the dynamic behavior is also influenced by the temperature-dependent parameters of the medium. When the thermometers are installed in air, for example, the heat transfer coefficient α decreases with increasing temperature, owing to the temperature-dependent material data of the air, at constant speed *v*. At the same time, heat radiation effects are so strong that the heat transfer improves despite the decreasing convective heat transfer coefficient. In this paper, a number of examples are used to establish a model for the temperature dependence of the dynamic parameters for various thermometer designs. Both numerically and experimentally determined results for the determination of the dynamic characteristic values are included in the consideration.

## 1. Introduction

In the 1980s and 1990s, several works were published dealing with the temperature dependence of the dynamic behavior of contact thermometers [1,2,3]. The authors had been working on this subject for several years [4,5,6]. To evaluate the dynamic behavior of contact thermometers quantitatively, dynamic parameters (time percentage values *t*_x_, time constants *τ*, or cut-off frequencies *f*_G_) are used. They can be described as both changing the medium temperature of the process and by generating a step response when the temperature sensor changes from one medium with the temperature *T*_1_ to another medium with the temperature *T*_2_ (*T*_1_ ≠ *T*_2_). In previous standards for contact thermometers for the determination of the dynamic behavior, the recording of step responses by Δ*T* ≈ 20–40 K in water or air was prescribed. However, conclusions cannot always be drawn from the obtained characteristic values about the dynamic behavior under other conditions (e.g., when using thermocouples in the hot steam range or in the exhaust gas systems of vehicles (temperatures up to 1100 °C)). The characteristic values of sensors at such high temperatures were not determined by using the equipment of the Institute for Process Measurement and Sensor Technology at the TU Ilmenau. Therefore, numerical calculations were carried out. At the beginning, a simple wire-wound measuring resistor was considered for these numerical calculations since only the temperature dependence of the material data of Al_2_O_3_ needs to be taken into account, and analytical results can be used for the comparison of the numerically calculated ones [6]. Only theoretically determined results were described in [6], so in the present paper analytical, numerical, and experimentally determined results are presented.

**Figure 1:**
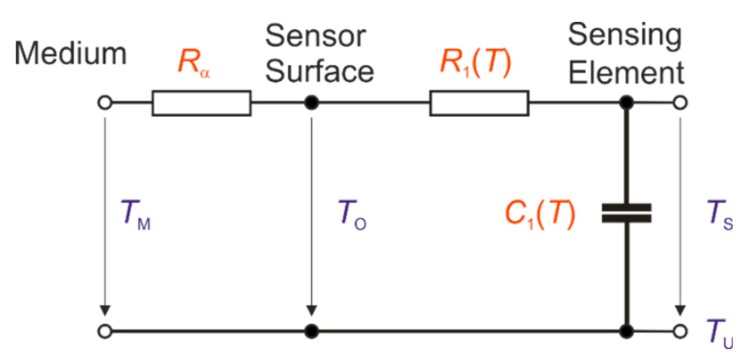
Electrical analogy model of the first-order time delay element of a sensor (cylinder). where:*R*_α_—thermal resistance caused by convection;*R*_1_—internal thermal resistance of the sensor caused by conduction;*C*_1_—heat capacity of the sensor;*T*_M_—medium temperature;*T*_O_—temperature of the sensor surface;*T*_S_—sensor temperature;*T*_U_—ambient temperature.

For the analytical calculation, the dynamic behavior of a sensing element (ceramic cylinder) can be explained using an electrical analogy model of a first-order time delay element (Figure 1).

The time constant *τ* can be calculated by:

(1) $$\tau =C_{1}\cdot \left(R_{\alpha }+R_{1}\right)=V\cdot \rho \cdot c\cdot \left(\frac{1}{\alpha \cdot A_{M}}+\frac{1}{2\cdot \pi \cdot l\cdot \lambda }\cdot \ln\frac{r_{a}}{r_{i}}\right)$$

where:

*V*—volume of the sensing element;
*ρ*—density of the sensor;
*c*—specific heat capacity of the sensor;
*α*—heat transfer coefficient by convection;
*A*_M_—sensor surface;
*l*—length of the sensor;
*λ*—thermal conductivity of the sensor;
*r*_a_*, r*_i_—outer and inner radius of the sensor.

The time constant *τ* is proportional to the inverse thermal diffusivity *c*·*ρ*/*λ* as well as to *c*/*λ* if the density ρ is constant [6]. ANSYS software (mechanical APDL 17) was used for numerical calculations (finite element analysis). 

In this paper, the theoretically obtained results are compared to real thermometers, with experiments carried out using the test equipment of the Institute for Process Measurement and Sensor Technology.

## 2. Test Equipment

**Figure 2:**
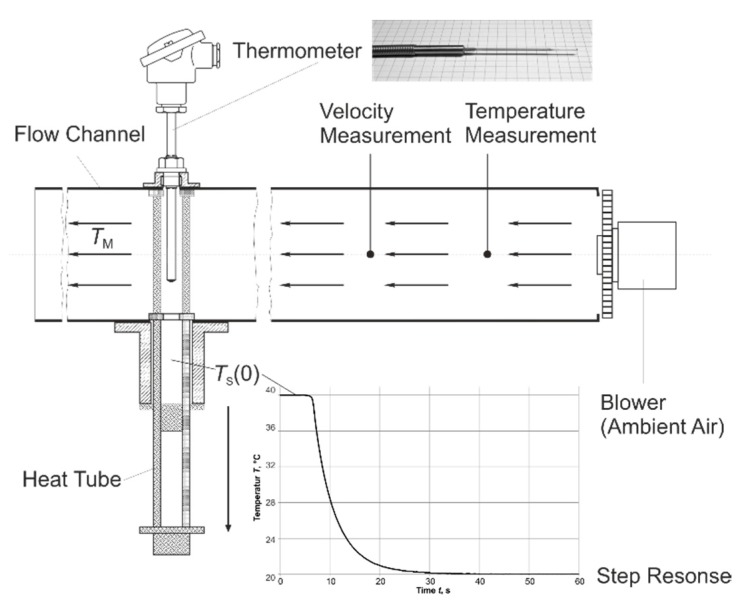
Schematic set-up for the experimental determination of the dynamic behavior of thermometers.

For the experimental determination of the dynamic behavior, step responses were applied with thermometers using the test equipment of the Institute for Process Measurement and Sensor Technology. This equipment is based on publications of F. Lieneweg [7], and consists of an air flow channel and a heat tube. The thermometers can be heated to a temperature of *T*_S_(0) = 200 °C using a heat tube. At the beginning of the step, the tube drops down, driven by gravity, and the thermometer is cooled by forced convection in ambient air with different velocities between 1 m∙s^−1^ and 10 m∙s^−1^ (Figure 2).

The time-percent values *t*_x_ were calculated by a normalized step response [8]:

(2) h(tx)=TS(tx)−TS(0)TM−TS(0)=1−e−tτ

where:

*T*_S_(*t*_x_)—temperature by time *t*_x_;
*T*_S_(0)—temperature at the beginning of the step (*t* = 0 s);
*T*_M_—temperature of the medium (in this case: air).

In [9], the authors described the influences of the measurement uncertainty of the test equipment. 

The following influencing factors must be considered when determining the flow velocity:

(3) $$v_{L}=v_{M}+\Delta v_{S}+\Delta v_{MS}+\Delta v_{SP}$$

where:

*v*_L_—air velocity;
*v*_M_—measured velocity;
Δ*v*_S_—uncertainty of the velocity-measuring sensor;
Δ*v*_MS_—difference between the velocity measurement and the velocity at measuring point;
Δ*v*_SP_—influence of an inhomogeneous velocity profile.

The measurement uncertainty in the determination of the time-percent values can be estimated with the help of the following equation:

(4) $$\Delta \left(t_{x}\right)=\frac{\Delta \left(h\left(t_{x}\right)\right)}{S\left(h\left(t_{x}\right)\right)}+\Delta \left(t_{A}\right)+\Delta \left(t_{MG}\right)+\Delta \left(t_{MSU}\right)+\Delta \left(t_{Fall}\right),$$

where:

Δ(*t*_x_)—uncertainty of the respective time-percent value;
Δ(*h*(*t*_x_))—uncertainty in determining the normalized temperature;
*S*(*h*(*t*_x_))—increase of the respective time-percent value;
Δ(*t*_A_)—uncertainty of the sampling time;
Δ(*t*_MG_)—uncertainty of the measuring device (HP 34410A);
Δ(*t*_MSU_)—uncertainty of the measuring switch (PREMA 2024);
Δ(*t*_Fall_)—uncertainty by falling of the heat tube.

These individual contributions in [9] are presented as examples. With these uncertainties, the time-percent values can be specified for the individual measurements.

## 3. Comparison of Analytical, Numerical, and Experimental Results for an Existing Sensor Element

**Figure 3:**
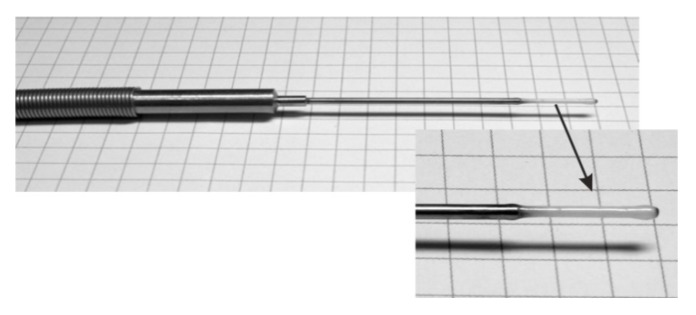
Special thermometer with an unshielded sensing element.

For measurements at room temperature, a special thermometer with an unshielded sensing element was built (Figure 3). This sensor has a diameter *d* = 1 mm and a length *l* = 15 mm, and the material is Al_2_O_3_.

This sensor was very well suited for simulating the dynamic behavior of a first-order time-delay element. Numerical calculations were first performed to determine the dependence of the dynamic characteristic values on the temperature. Only the sensor element itself (without the support) was modeled.

Here, axial-symmetrical elements were used as geometric models for the cylinder. The temperature dependencies of the specific heat capacity *c* and the thermal conductivity *λ* were transferred using a spreadsheet with temperatures from 0 to 1000 °C in increments of 100 K [10,11]. The density of the material was assumed to be constant with a value of *ρ* = 3900 kg·m^−3^. The inverse thermal diffusivity *a* ($a^{-1}=\frac{c\cdot \rho }{\lambda }$) increased with rising temperature for the material used.

**Figure 4:**
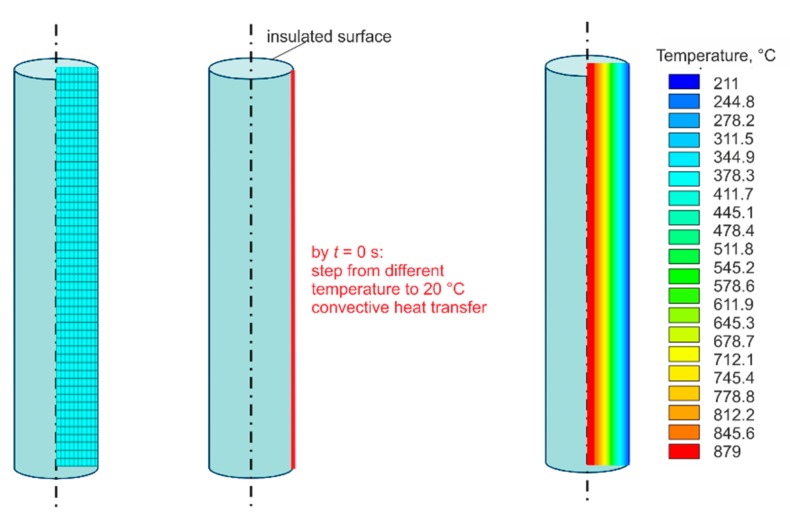
Finite element analysis (FEA) model, boundary condition, and an example of typical temperature gradient field.

The cooling from different starting temperatures (see markers in Figure 4) to room temperature were calculated. At the beginning of the temperature step (*t* = 0 s), a convective heat transfer coefficient *α* = 171 W∙m^−2^·K^−1^ and ambient air temperature *T* = 20 °C were set as the boundary condition at the right line of the axial-symmetrical model and all other surface lines were insulated (Figure 4).

The convective heat transfer coefficient *α* decreased with increasing temperature, and thermal radiation was not considered. The respective step responses to the end time of 120 s were calculated with automatically selected time steps between 10^−8^ and 0.05 s.

**Figure 5:**
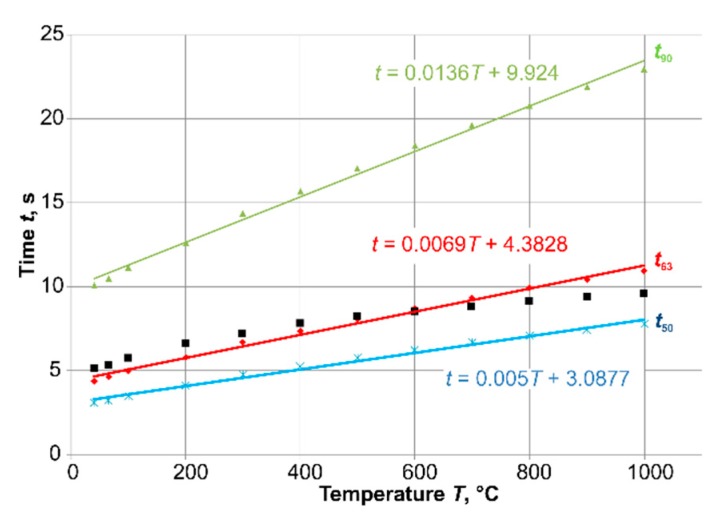
FEA results for steps from different temperatures to *T* = 20 °C.

The time-percent values (the time at which a certain percentage value of the transition function is reached) based on these step responses are shown in Figure 5. The calculated results (*t*_50_: blue line, *t*_63_: red line, and *t*_90_: green line) confirm the assumption that the time-percent values also increased with increasing temperature, but in this case the increase was very slight.

For first-order time-delay elements, the time constant *τ* corresponds to the time percentage value *t*_63_. To compare this value with the value of the time constant *τ*, the time constant was calculated analytically (see Equation (1), Section 1) according to [8]. The black points in Figure 5 show the analytically calculated results of the time constant *τ*. Up to a starting temperature of 400 °C there was good congruence with the values of *t*_63_.

**Figure 6:**
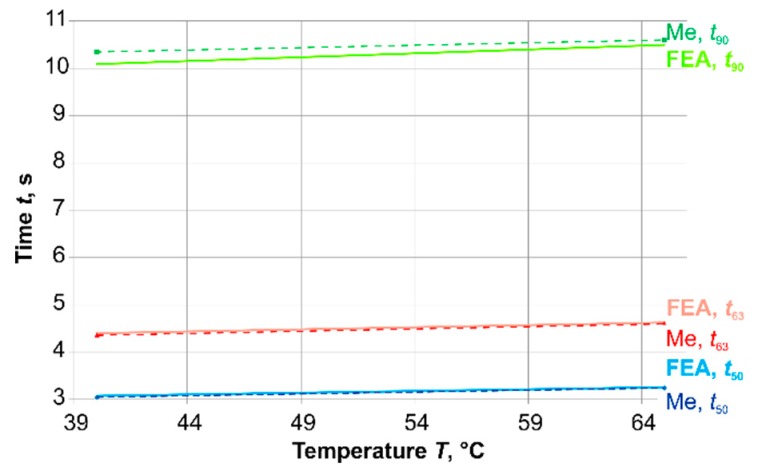
Comparison of FEA and experimental results (Me).

The results were compared with experiments for steps from *T* = 40 °C and *T* = 65 °C (Figure 6). Due to the design and the material data of the sensor, the experiments could only be carried out in this temperature range. The results in Figure 6 show a good correlation between the calculated and the measured time-percent values in this small temperature range. 

## 4. Investigations with Typical Industrial Thermometers

**Figure 7:**
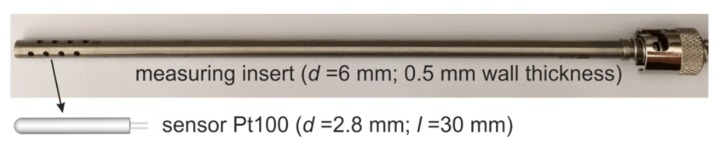
Industrial resistance thermometer used.

Firstly, the dynamic behavior of a simple sensor element at higher temperatures was investigated. Later, another sensor was mounted in a measuring insert as protection against higher air velocities (Figure 7). The measuring insert has holes at its tip to shorten the time constant compared to conventional measuring inserts.

The thermometer was analyzed in the test equipment at the starting temperatures *T*_S_ (0) = 40–200 °C. The time-percent values were determined as the mean values of five step responses per temperature. Afterwards, these results were compared with FEA calculations. Only the sensor without a measuring insert was modeled for these calculations, as described in Section 3. The temperature-dependent material parameters of Al_2_O_3_ were the same. The convective heat transfer coefficient was larger than the value in Section 2 due to the measuring insert. It changed with temperature in a range of *α* = 84.13–85.57 W∙m^−2^·K^−1^. The radiation between the thermometer and the surrounding area was considered. The step response was simulated for *t* = 400 s, with automatically selected time steps between 10^−6^ s and 0.05 s.

**Figure 8:**
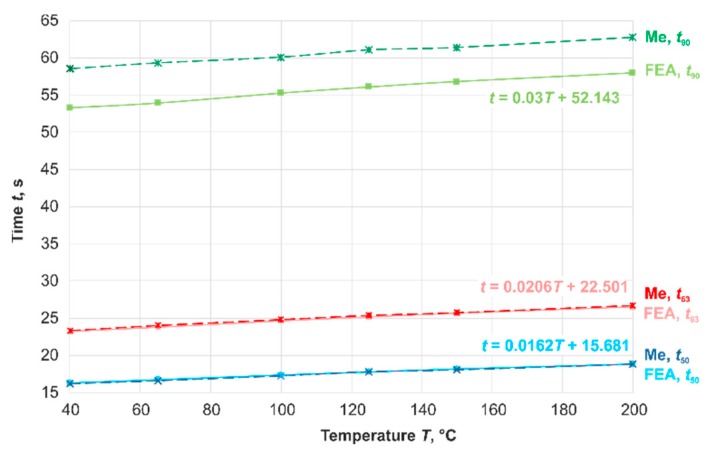
Comparison of the results of FEA calculation and measurement (Me) for the industrial resistance thermometer shown in Figure 7.

The results show the temperature dependence of dynamic parameters and a very good agreement between the calculated values and the measured ones for *t*_50_ and *t*_63_. But, in this simulation, the measuring insert, the influence of a differently temperature-controlled environment, and the place of installation were not considered. Therefore, there is a difference between the results of FEA-calculation (green line) and measurement (green dashed line) for the time-percent value *t*_90_ (Figure 8).

In all the previously described cases, the sensor was assumed to be made of one material. However, what would happen if the sensor and the thermometer consisted of more than one component with various material properties? 

**Figure 9:**
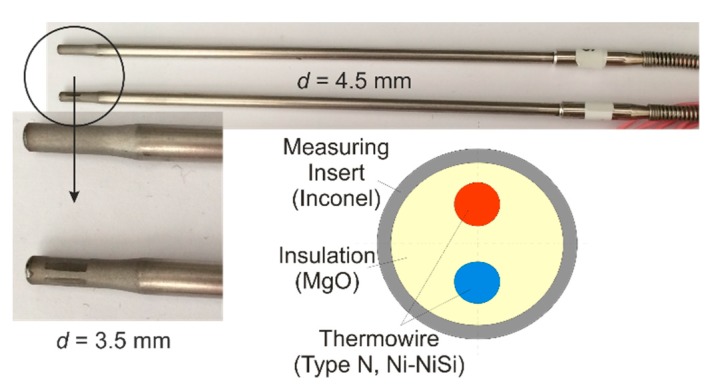
Thermocouple type N.

To answer this question, a typical industrial sheathed thermocouple was used (Figure 9).

**Figure 10:**
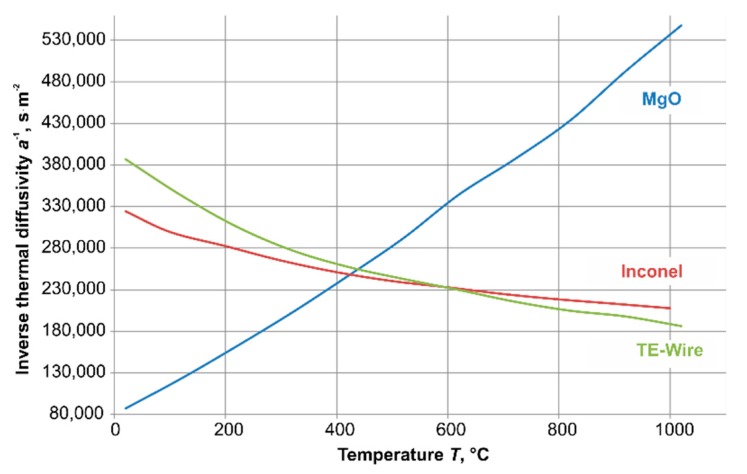
Inverse thermal diffusivity *a*^−1^ of the materials used [10,12,13].

The temperature dependence of the inverse thermal diffusivity *a*^−1^ was different for the three materials used—it increased with rising temperature for MgO, and it decreased with rising temperature for the two metals (Figure 10).

**Figure 11:**
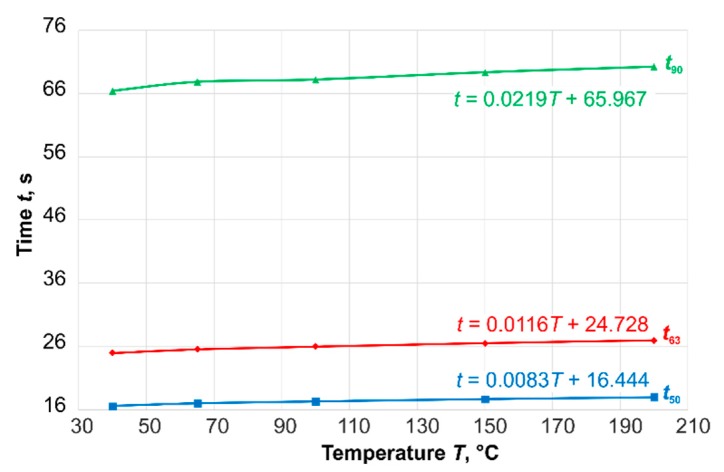
Experimental results (air, steps from various temperature to ambient air, *v* = 3 m∙s^−1^).

The experimental data using this thermocouple in the test equipment (Figure 2) also showed a dependency of the dynamic characteristic values on the temperature (Figure 11).

However, this was less than (approximately half as large as) the measurement of the resistance thermometer, which can be explained by the fact that the volume of the insulation ceramic was about twice the volume of the thermocouple wires and the measuring insert. The increase in the thermal diffusivity of the ceramic with rising temperature was more pronounced than the decrease in the thermal diffusivity of the metals in response to rising temperature. The thermocouples used in the experiments were developed for optimal control and safety value monitoring of the combustion process in engines—especially car engines. Thermocouples used in the exhaust systems of combustion engines are exposed to high temperature gradients, temperature steps (Δ*T* > 900 K), high air flow velocities, and pressure. A hot gas channel was built to investigate the dynamic behavior of these thermometers [14]. In the forthcoming months, the thermocouple will be measured in this hot gas channel in order to evaluate how the dynamic characteristic values behave under these conditions.

## 5. Conclusions

It was possible to verify the temperature dependence of dynamic parameters of various thermometers through numerical calculations and the measurements obtained by using different test equipment. For ceramic sensing resistors, a linear correlation between dynamic parameters and the inverse thermal diffusivity of the sensor material was found.

Generally, the dynamic parameters depend on:

- Temperature-dependent material properties of medium and thermometer;
- The thermometer design and installation conditions;
- Heat transfer conditions;
- Surrounding area.

Regarding the relation between thermometer design and the materials employed (in terms of thermal resistance and capacity), the boundary conditions (particularly heat transfer coefficient), the installation conditions, etc., are decisive. Therefore, predictions cannot be made easily and a simple analytical model for the relation between the material parameters and the dynamic behavior of industrial thermometers has not yet been formulated.
